# Carijoside A, a Bioactive Sterol Glycoside from an Octocoral *Carijoa* sp. (Clavulariidae)

**DOI:** 10.3390/md8072014

**Published:** 2010-06-29

**Authors:** Chih-Yang Liu, Tsong-Long Hwang, Mei-Ru Lin, Yung-Husan Chen, Yu-Chia Chang, Lee-Shing Fang, Wei-Hsien Wang, Yang-Chang Wu, Ping-Jyun Sung

**Affiliations:** 1 Department of Life Science and Graduate Institute of Biotechnology, Graduate Institute of Marine Biotechnology, National Dong Hwa University, Checheng, Pingtung 944, Taiwan; E-Mails: belle_ames@hotmail.com (C.-Y.L.); jay0404@gmail.com (Y.C.C.); 2 National Museum of Marine Biology and Aquarium, Checheng, Pingtung 944, Taiwan; E-Mails: linmeiru@hotmail.com (M.R.L.); tony_chen72001@yahoo.com.tw (Y.H.C.); 3 Graduate Institute of Natural Products, Chang Gung University, Taoyuan 333, Taiwan; E-Mail: htl@mail.cgu.edu.tw (T.L.H.); 4 Department of Sport, Health, and Leisure, Cheng Shiu University, Kaohsiung 833, Taiwan; E-Mail: lsfang@csu.edu.tw (L.S.F.); 5 Department of Marine Biotechnology and Resources, Asia-Pacific Ocean Research Center, National Sun Yat-sen University, Kaohsiung 804, Taiwan; E-Mail: whw@nmmba.gov.tw (W.H.W.); 6 Graduate Institute of Natural Products, Kaohsiung Medical University, Kaohsiung 807, Taiwan

**Keywords:** Carijoa, carijoside, glycoside, superoxide anion, elastase, cytotoxicity

## Abstract

A new bioactive sterol glycoside, 3*β*-*O*-(3*′*,4*′*-di-*O*-acetyl-*β*-d-arabinopyranosyl) -25*ξ*-cholestane-3*β*,5*α*,6*β*,26-tetrol-26-acetate) (carijoside A, **1**), was isolated from an octocoral identified as *Carijoa* sp. The structure of glycoside **1** was established by spectroscopic methods and by comparison with spectral data for the other known glycosides. Carijoside A (**1**) displayed significant inhibitory effects on superoxide anion generation and elastase release by human neutrophils and this compound exhibited moderate cytotoxicity toward DLD-1, P388D1, HL-60, and CCRF-CEM tumor cells.

## 1. Introduction

**Figure 1:**
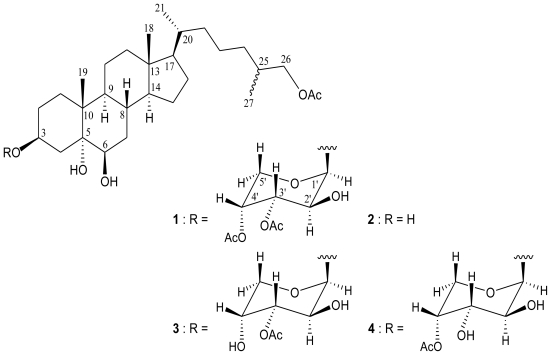
The Structures of Carijoside A (**1**), Cholestane-3*β*,5*α*,6*β*,26-tetrol-26-acetate (**2**), Riisein A (**3**) and Riisein B (**4**).

Previous studies on the chemical constituents from the octocorals belonging to the genus *Carijoa* (=*Telesto*) have yielded a series of bioactive substances including amide [1], steroid [1–4], and prostanoid analogs [3,5]. In our continuing studies on the chemical constituents of octocorals distributed in Taiwan waters, a new sterol glycoside, carijoside A (**1**) (Figure 1) was isolated from an octocoral identified as *Carijoa* sp. The structure of **1** was determined by spectroscopic methods and by comparison of spectral data with those of known sterols. In this paper, we describe the isolation, structure determination, and bioactivity of glycoside **1**.

## 2. Results and Discussion

**Figure 2:**
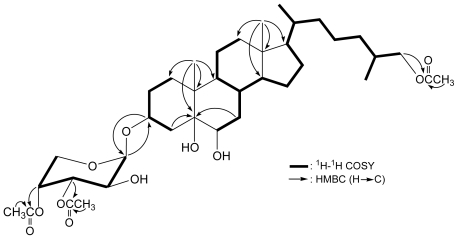
The ^1^H-^1^H COSY and Selective Key HMBC Correlations of **1**.

Carijoside A (**1**) was isolated as a white powder. The molecular formula of **1** was established as C_38_H_62_O_11_ (eight degrees of unsaturation) from a sodiated molecule at *m/z* 717 in ESIMS and further supported by HRESIMS (*m/z* 717.4186, calcd. 717.4190, [C_38_H_62_O_11_Na]^+^). The IR spectrum of **1** showed bands at 3466 and 1743 cm^−1^, consistent with the presence of hydroxy and ester carbonyl groups. Analysis of 2D NMR experiments revealed that carijoside A (**1**) was a pentose glycoside derivative of a known trihydroxy sterol, cholestane-3*β*,5*α*,6*β*-tetrol-26-acetate (=25*ξ*-cholestane-3*β*,5*α*,6*β*,26-tetrol-26-acetate) (**2**), a cytotoxic steroid previously isolated from *Telesto riisei*, collected from Northeast Pass, Chuuk, Federated States of Micronesia [1] and Mangaratiba, Rio de Janeiro State, Brazil [4]. In addition to the pentose moiety, the ^13^C NMR and DEPT spectra of **1** showed that this compound has 29 carbons for the cholestane carbon with an acetoxy group (Table 1), including five methyls, 12 *sp*^3^ methylenes, eight *sp*^3^ methines, three *sp*^3^ quaternary carbons, and an *sp*^2^ quaternary carbon. From the ^13^C and ^1^H NMR spectra (Table 1), the presence of four oxygenated C atoms at *δ*_C_ 74.9 (d, CH-3), 75.9 (s, C-5), 76.0 (d, CH-6), and 69.6 (t, CH_2_-26) in the ^13^C NMR spectrum and two oxymethine protons at *δ*_H_ 4.07 (1H, m, H-3) and 3.54 (1H, br s, H-6) and a pair of oxygen-bearing methylene protons at *δ*_H_ 3.84 (1H, m) and 3.97 (1H, m) in the ^1^H NMR spectrum were determined. From the ^1^H-^1^H COSY spectrum, several different structural units, including C-1/-2/-3/-4, C-6/-7/-8/-9/-11/-12, C-8/-14/-15/-16/-17/-20/-22/-23/-24/-25/-26(-27), and C-20/-21, were identified (Figure 2), which were assembled with the assistance of an HMBC experiment permitted elucidation of the cholestane carbon skeleton of **1**. The ring junctions C-18 and C-19 methyl groups were positioned at C-13 and C-10 from the HMBC correlations between H_3_-18/C-12, -13, -14, -17 and H_3_-19/C-1, -5, -9, -10, respectively (Figure 2). An oxymethine unit at *δ*_C_ 76.0 correlated to the methine proton at *δ*_H_ 3.54 in the HMQC spectrum, proving the attachment of a hydroxy group at C-6. The remaining hydroxy and acetoxy groups at C-5 and C-26 in the cholestane moiety of **1** were indicated by analysis of HMBC correlations and characteristic NMR signals. However, the doubling of the 26-acetoxymethylene and H_3_-27 signals indicate that **1** consists of a stereoisomeric mixture (25*R*/25*S*).

Furthermore, the proton NMR signals for the pentose pyranoside between *δ*_H_ 3.6–5.3 and by the corresponding ^13^C NMR signals between *δ*_C_ 60–71 and for the characteristic sugar anomeric carbon (*δ*_C_ 97.4) and its corresponding methine proton (*δ*_H_ 5.06) (Tables 1 and 2). ^1^H NMR coupling constant analysis of the pyranose ring indicated the presence of a pyranoarabinoside sugar linked to the sterol by a *β*-glycoside linkage. The attachment of the sugar moiety at C-3 in **1** was based on the key HMBC correlations (Figure 2). The sugar anomeric carbon C-1*′* (*δ*_C_ 97.4) and the aglycon carbon C-3 (*δ*_C_ 74.9) showed correlations with H-3 (*δ*_H_ 4.07) and H-1*′* (*δ*_H_ 5.06), respectively. NMR data also indicated the presence of two additional acetate esters positioned at C-3*′* (*δ*_H_ 5.13, 1H, dd, *J* = 10.4, 3.2 Hz; *δ*_C_ 70.5, CH) and C-4*′* (*δ*_H_ 5.25, 1H, br s; *δ*_C_ 69.4, CH). Based on detailed analysis, the structure of **1** was found to be similar with those of two known cytotoxic sterol glycosides, riisein A (3*β*-*O*-(3*′*-*O*-acetyl-*β*-d-arabinopyranosyl)- 25*ξ*-cholestane-3*β*,5*α*,6*β*,26-tetrol-26-acetate) (**3**) and riisein B (3*β*-*O*-(4*′*-*O*-acetyl-*β*-d-arabinopyranosyl)-25*ξ*-cholestane-3*β*,5*α*,6*β*,26-tetrol-26-acetate) (**4**) [4], with the exception that the 4*′*-hydroxy group in **3** and 3*′*-hydoxy group in **4** was replaced by an acetoxy group in **1**.

In anti-inflammatory activity testing, glycoside **1** displayed significant inhibitory effects on superoxide anion generation (IC_50_ = 1.8 μg/mL) and elastase release (IC_50_ = 6.8 μg/mL) by human neutrophils [6,7] and this compound exhibited moderate cytotoxicity towards DLD-1 (human colon adenocarcinoma), P388D1 (murine macrophage cells), HL-60 (human premyelocytic leukemia), and CCRF-CEM (human T-cell acute lymphoblastic leukemia) tumor cells (ED_50_ = 9.7, 10.4, 12.0, and 13.1 μg/mL), respectively [8].

## 3. Experimental Section

### 3.1. General Experimental Procedures

Melting points were measured on a FARGO apparatus and were uncorrected. Optical rotation values were measured with a JASCO P-1010 digital polarimeter. The Infrared spectra were obtained on a VARIAN DIGLAB FTS 1000 FT-IR spectrophotometer. The NMR spectra were recorded on a VARIAN MERCURY PLUS 400 FT-NMR at 400 MHz for ^1^H and 100 MHz for ^13^C, in CDCl_3_, respectively, Proton chemical shifts were referenced to the residual CHCl_3_ signal (*δ*_H_ 7.26 ppm). ^13^C NMR spectra were referenced to the center peak of CDCl_3_ at *δ*_C_ 77.1 ppm. ESIMS and HRESIMS data were recorded on a BRUKER APEX II mass spectrometer. Gravity column chromatography was performed on silica gel (230–400 mesh, Merck, Darmstadt, Germany). TLC was carried out on precoated Kieselgel 60 F_254_ (0.2 mm, Merck) and spots were visualized by spraying with 10% H_2_SO_4_ solution followed by heating.

### 3.2. Animal Material

Specimen of the octocoral *Carijoa* sp. were collected off the coast of Pingtung county, Southern Taiwan, in August 2008, and this organism was identified by comparison with previous descriptions [9]. The voucher specimen was deposited in the National Museum of Marine Biology and Aquarium, Taiwan.

### 3.3. Extraction and Isolation

The freeze-dried and minced material of *Carijoa* sp. (wet weight 1588 g, dry weight 422 g) were extracted with a mixture of MeOH and CH_2_Cl_2_ (1:1). The extract was partitioned between EtOAc and H_2_O. The EtOAc layer was separated by silica gel and eluted using hexane/EtOAc (stepwise, 20:1–pure EtOAc) to yield 35 fractions. Fraction 17 was separated by silica gel and eluted using hexane/acetone (stepwise, 20:1–1:1) to afford **1** (1.6 mg, 2:1).

Carijoside A (**1**): white powder; mp 171–172 °C (decomp.); [*α*]_D_^22^-112 (*c* 0.06, CHCl_3_); IR (neat) *ν*_max_ 3466, 1743 cm^−1^; ^1^H NMR (CDCl_3_, 400 MHz) and ^13^C NMR (CDCl_3_, 100 MHz) data, see Table 1; ESIMS *m/z* 717 (M + Na)^+^; HRESIMS *m/z* 717.4186 (calcd for C_38_H_62_O_11_Na, 717.4190).

### 3.4. Human Neutrophil Superoxide Anion Generation and Elastase Release

Human neutrophils were obtained by means of dextran sedimentation and Ficoll centrifugation. Superoxide anion generation was carried out according to procedures described previously [10,11]. Briefly, superoxide anion production was assayed by monitoring the superoxide dismutase-inhibitable reduction of ferricytochrome *c*. Elastase release experiments were performed using MeO-Suc-Ala-Ala-Pro-Val-*p*-nitroanilide as the elastase substrate.

### 3.5. Cytotoxicity Testing

The cytotoxicity of tested compound **1** was assayed using a modification of the MTT [3-(4,5-dimethylthiazol-2-yl)-2,5-diphenyltetrazolium bromide] colorimetric method. Cytotoxicity assays were carried out according to the procedures described previously [12,13].

## Figures and Tables

**Table 1:** ^1^H and ^13^C NMR Data for Steroids **1** and **2**.

C/H	1		2^a^	
	^1^H^b^/*δ*	^13^C^c^/*δ*	^1^H^d^/*δ*	^13^C^e^/*δ*
1	1.42, m; 1.60 m	32.2 (CH_2_)	1.40 m; 1.52 m	32.3 (CH_2_)
2	1.86 m (2H)	28.6 (CH_2_)	1.86 m	30.7 (CH_2_)
3	4.07 m	74.9 (CH)	4.08 m	67.6 (CH)
4	1.69 m; 2.10 m	37.3 (CH_2_)	1.63 m; 2.08 m	40.6 (CH_2_)
5		75.9 (C)		76.0 (C)
6	3.54 br s	76.0 (CH)	3.52 br s	75.8 (CH)
7	1.59 m (2H)	34.6 (CH_2_)	1.60 m	34.3 (CH_2_)
8	1.74 m	30.1 (CH)	1.72 m	30.2 (CH)
9	1.16 m	45.9 (CH)	1.25^j^	45.7 (CH)
10		38.4 (C)		38.2 (C)
11	1.38 m (2H)	21.1 (CH_2_)	1.37 m	21.1 (CH_2_)
12	1.16 m; 2.02 m	39.9 (CH_2_)	1.16 m; 1.98 m	39.9 (CH_2_)
13		42.7 (C)		42.7 (C)
14	1.08 m	55.9 (CH)	1.08 m	55.9 (CH)
15	1.08 m; 1.59 m	24.1 (CH_2_)	1.08 m; 1.56 m	24.1 (CH_2_)
16	1.24 m; 1.77 m	28.2 (CH_2_)	1.22 m; 1.83 m	28.2 (CH_2_)
17	1.10 m	56.2 (CH)	1.11 m	56.2 (CH)
18	0.68 s	12.1 (CH_3_)	0.67 s	12.1 (CH_3_)
19	1.20 s	17.0 (CH_3_)	1.17 s	17.0 (CH_3_)
20	1.36 m	35.7 (CH)	1.37 m	36.0 (CH)
21	0.90 d (6.0)^f^	18.7 (CH_3_)	0.92 d (7.0)	18.6 (CH_3_)
22	1.00 m; 1.33 m	36.0 (CH_2_)	1.00 m; 1.37 m	35.7 (CH_2_)
23	1.33 m (2H)	23.3 (CH_2_)	1.37 m	23.3 (CH_2_)
24	1.13 m; 1.27 m^g^	33.9 (CH_2_)	1.72 m	33.9 (CH_2_)
		33.7		33.7
25	1.76 m	32.5 (CH)	1.77 m	32.5 (CH)
		32.5		32.4
26a	3.82 dd (10.4, 1.6)	69.6 (CH_2_)	3.82 dd (2.5, 10.5)	69.9 (CH_2_)
	3.85 dd (10.4, 1.6)	69.4	3.84 dd (2.5, 10.5)	69.5
b	3.92 dd (10.4, 6.0)^h^		3.93 dd (6.0, 10.5)	
	3.93 dd (10.4, 6.0)^h^		3.95 dd (6.0, 10.5)	
27	0.92 d (6.8)	16.9 (CH_3_)	0.90 d (6.0)	16.8 (CH_3_)
	0.91 d (6.8)	16.8	0.91 d (6.0)	
26-OAc		171.3 (C)		171.3 (C)
	2.05 s	20.9 (CH_3_)^i^	2.05 s	21.0 (CH_3_)
1*′*	5.06 d (4.0)	97.4 (CH)		
2*′*	3.93 dd (10.4, 4.0)	67.3 (CH)		
3*′*	5.13 dd (10.4, 3.2)	70.5 (CH)		
4*′*	5.25 br s	69.4 (CH)		
5*′*	3.65 dd (12.8, 2.0); 3.97 m^h^	60.9 (CH_2_)		
3*′*-OAc		170.9 (C)		
	2.07 s	21.0 (CH_3_)^i^		
4*′*-OAc		170.4 (C)		
	2.13 s	21.0 (CH_3_)^i^		

a: Data was reported by Maia *et al.* [4];

b: Spectrum recorded at 400 MHz in CDCl_3_ at 25 °C;

c: Spectrum recorded at 100 MHz in CDCl_3_ at 25 °C;

d: Spectrum recorded at 500 MHz in CDCl_3_;

e: Spectrum recorded at 50 MHz in CDCl_3_;

f: *J* values (in Hz) in parentheses;

g: The ^1^H NMR data for these methylene protons were assigned by the assistance of Dept and HMQC spectra;

h: Signals overlapping;

i: Data exchangeable;

j: The coupling pattern for this methine proton was not assigned.

**Table 2:** NMR Data for the 3*′*- and 4*′*-*O*-Acetyl-arabinopyranoside Components in Glycosides Carijoside A (**1**), Riisein A (**3**) and Riisein B (**4**).

C/H	1		3^a^		4^a^	
	^1^H/*δ*	^13^C/*δ*	^1^H/*δ*	^13^C/*δ*	^1^H/*δ*	^13^C/*δ*
1*′*	5.06 d (4.0)	97.4 (CH)	5.04 d (4.5)	97.8 (CH)	5.01 d (3.5)	97.6 (CH)
2*′*	3.93 dd (10.4, 4.0)	67.3 (CH)	3.94 m	67.3 (CH)	3.80 dd (3.5, 10.5)	68.8 (CH)
3*′*	5.13 dd (10.4, 3.2)	70.5 (CH)	5.07 dd (3.0, 9.8)	73.1 (CH)	3.94 m	67.8 (CH)
4*′*	5.25 br s	69.4 (CH)	4.03 br s	68.3 (CH)	5.15 br s	71.1 (CH)
5*′*	3.65 dd (12.8, 2.0)	60.9 (CH_2_)	3.66 dd (2.0, 12.5)	62.6 (CH_2_)	3.68 br d (11.0)	60.5 (CH_2_)
	3.97 m		3.94 m		3.93 m	
3*′*-OAc		170.9 (C)		170.9 (C)		
	2.07 s	21.0 (CH_3_)	2.17 s	21.2 (CH_3_)		
4*′*-OAc		170.4 (C)				170.9 (C)
	2.13 s	21.0 (CH_3_)			2.17 s	21.2 (CH_3_)

a: Data was reported by Maia *et al.* [4].
